# Deficiency of Transcription Factor Brn4 Disrupts Cochlear Gap Junction Plaques in a Model of DFN3 Non-Syndromic Deafness

**DOI:** 10.1371/journal.pone.0108216

**Published:** 2014-09-26

**Authors:** Yoshinobu Kidokoro, Keiko Karasawa, Osamu Minowa, Yoshinobu Sugitani, Tetsuo Noda, Katsuhisa Ikeda, Kazusaku Kamiya

**Affiliations:** 1 Juntendo University Faculty of Medicine, Department of Otorhinolaryngology, Tokyo, Japan; 2 BioResource Center, Institute of Physical and Chemical Research (RIKEN), Tsukuba, Japan; 3 Department of Cell Biology, Japanese Foundation for Cancer Research, Cancer Institute, Tokyo, Japan; Albert Einstein College of Medicine, United States of America

## Abstract

*Brn4*, which encodes a POU transcription factor, is the gene responsible for DFN3, an X chromosome–linked, non-syndromic type of hearing loss. Brn4-deficient mice have a low endocochlear potential (EP), hearing loss, and ultrastructural alterations in spiral ligament fibrocytes, however the molecular pathology through which Brn4 deficiency causes low EP is still unclear. Mutations in the Gjb2 and Gjb6 genes encoding the gap junction proteins connexin26 (Cx26) and connexin30 (Cx30) genes, respectively, which encode gap junction proteins and are expressed in cochlear fibrocytes and non-sensory epithelial cells (i.e., cochlear supporting cells) to maintain the proper EP, are responsible for hereditary sensorineural deafness. It has been hypothesized that the gap junction in the cochlea provides an intercellular passage by which K^+^ is transported to maintain the EP at the high level necessary for sensory hair cell excitation. Here we analyzed the formation of gap junction plaques in cochlear supporting cells of Brn4-deficient mice at different stages by confocal microscopy and three-dimensional graphic reconstructions. Gap junctions from control mice, which are composed mainly of Cx26 and Cx30, formed linear plaques along the cell-cell junction sites with adjacent cells. These plaques formed pentagonal or hexagonal outlines of the normal inner sulcus cells and border cells. Gap junction plaques in Brn4-deficient mice did not, however, show the normal linear structure but instead formed small spots around the cell-cell junction sites. Gap junction lengths were significantly shorter, and the level of Cx26 and Cx30 was significantly reduced in Brn4-deficient mice compared with littermate controls. Thus the Brn4 mutation affected the assembly and localization of gap junction proteins at the cell borders of cochlear supporting cells, suggesting that Brn4 substantially contributes to cochlear gap junction properties to maintain the proper EP in cochleae, similar to connexin-related deafness.

## Introduction

Congenital sensorineural hearing loss occurs in 1 out of every 1000 births and is one of the most common congenital diseases. Deafness type 3 (DFN3) is the most common type of in X chromosome–linked, non-syndromic hearing loss. DFN3 is caused by mutations in Brn4, which is the gene that encodes the POU domain, class 3, transcription factor 4 (POU3F4) [1]. During embryogenesis, Brn4 is expressed in mesenchymal cells of the inner ear [2]. Brn4 is also associated with neuronal development, as radial bundle fasciculation and synapse formation are disrupted when POU3F4 is deleted from the otic mesenchyme [3]. This neuronal defect does not, however, explain the pathophysiology of the severe hearing loss that is present in Brn4-deficient mice and that is caused by the decreased EP [4].

To investigate the function of Brn4 in the cochlea, we developed Brn4-deficient mice as a model of DFN3 non-syndromic deafness [4]. Although, these mice show no gross morphological changes in their cochleae, there are severe ultrastructural alterations in the cochlear spiral ligament fibrocytes, which might have caused the decrease in EP that resulted in severe hearing loss [4]. This suggests that the initial pathology of Brn4 deficiency may be related to an impairment of intercellular junctions including gap junctions.

Cochlear fibrocytes of the mesenchymal non-sensory regions express gap junction proteins such as Cx26 and Cx30 and have important roles in the cochlear physiology of hearing, including the transport of potassium ions to generate an endocochlear potential in the endolymph, which is essential for the transduction of sound by hair cells. Cx26 and Cx30 are the two most abundantly expressed gap junction proteins in the cochlea, and both Cx26 and Cx30 are expressed at high levels in the supporting cells of the organ of Corti and fibrocytes in the spiral ligament [5]. Furthermore, co-immunostaining indicates that Cx26 and Cx30 are expressed in the same gap junction plaques (GJPs), and their co-assembly has been confirmed by co-immunoprecipitation of proteins extracted from cochlear tissues [5].

Mutations in the Cx26 and Cx30 genes are well known to be responsible for hereditary sensorineural deafness [6] [7], as are mutations in POU3F4 [1]. In this study, we analyzed the morphology, structure, or length formation of GJPs, the functional molecular complex of the gap junction, of Brn4-deficient mice at different stages by confocal microscopy and three-dimensional graphic reconstructions to clarify the relationship between the connexins and Brn4.

## Materials and Methods

### Animals and ethics statement

The care, maintenance, and treatment of animals in these studies followed protocols approved by the Institutional Animal Care and Use Committee at Juntendo University (Permit Number: 250192). 2–6 week old male mice were deeply anesthetized with an i.p. injection of Nembutal (50 mg/ml DAINIPPON SUMITOMO PHARMA) 0.5 mg per 10 g body weight before all examinations. POU3F4 mutant mice, in which the coding region was replaced with a pgk-neo (neomycin-resistance gene driven by the phosphoglycerate kinase gene promoter) cassette [4] were maintained in a C57BL/6 background. The absence of Brn4 protein was confirmed by immunohistochemistry with rabbit anti-POU3F4 (SIGMA) in inner ear frozen sections (data not shown). We minimized, to the extent possible, the number of animals used and their suffering.

### Immunohistochemistry

Mice were anesthetized and killed, and the inner-ear tissues were then removed. The cochleae were further dissected and fixed in 4% paraformaldehyde. Immunofluorescence staining with antibodies against Cx26 (mouse IgG; LifeSpan Biosciences) and Cx30 (rabbit IgG; Invitrogen) was performed on whole-mount preparations of the finely dissected organ of Corti, which included inner sulcus cells (ISCs). We incubated the tissues in the antibody solutions for 1 hour at room temperature after blocking (with 2% BSA in 0.01 M PBS). Fluorescence confocal images were obtained with a LSM510-META confocal microscope (Carl Zeiss). Cx30 was immunolabeled by red fluorescence, Cy3 (rabbit IgG; Sigma Aldrich), and observed by confocal laser microscopy using a 543-nm laser. Cx26 was immunolabeled by green fluorescence, Alexa488 (mouse IgG; Life Technologies), and observed by confocal microscopy using a 488-nm laser. Three-dimensional images were constructed with *z*-stacked confocal images by IMARIS (Bitplane). Quantitative analysis of the GJP length (mean ± S.E.M.) was measured with an LSM Image Browser (Zeiss), and data were compared using Student’s *t*-test (Microsoft Excel).

### Western blotting

Mouse cochlear proteins were extracted with T-PER Tissue Protein Extraction Reagent (Thermo Scientific) from at least six cochleae, which included the organ of Corti, lateral wall, and stria vascularis. The proteins were resolved by SDS–polyacrylamide gel electrophoresis (PAGE) with 4–20% mini-PROTEAN TGX gels (Bio-Rad Laboratories, Inc.) and then transferred onto a polyvinyl difluoride (PVDF) membrane (Amersham Hybond-P; GE Healthcare). After blocking, membranes were processed through sequential incubations with rabbit anti-Cx26 (1∶1000; Invitrogen), rabbit anti-Cx30 (1∶1000; Invitrogen) and mouse monoclonal anti–β-actin (1∶5000; Sigma) with horseradish peroxidase–conjugated anti–rabbit or anti–mouse IgG (1∶20000; GE Healthcare) as the secondary antibody. Amersham ECL Prime Western Blotting Detection Reagent (GE Healthcare) was then used for visualization, and the signal was developed on X-ray film (Amersham Hyperfilm ECL; GE Healthcare). Each experiment was repeated at least three times. The densitometric analysis of band intensities was performed with the NIH ImageJ software, and data were compared using Student’s *t*-test (Microsoft Excel).

### Auditory brainstem response (ABR)

All the recordings were performed in the soundproof chamber with electrostatic shield to avoid the electrical noise. Mice ranging in age from 2 weeks to 6 weeks were studied. For the ABR measurement, stainless-steel needle electrodes were placed at the vertex and ventrolateral to the left and right ears. The ABR was measured using the waveform storing and stimulus control of the Scope software from the Power Laboratory system (model PowerLab4/25; AD Instruments), and the electroencephalogram recording was made with an extracellular amplifier AC PreAmplifier (model P-55; Astro-Med). Acoustic stimuli were delivered to the mice through a coupler-type speaker (model ES1spc; Bio Research Center). The threshold was determined for frequencies of 8 and 20 kHz from a set of responses at varying intensities with 5-dB intervals, and the electrical signals were averaged at 512 repetitions. The maximum output level was 93.5 and 83.4 dB at 8 and 20 kHz, respectively, and 5 dB was added to these values when the ABR was not detected in the maximum output for statistical analysis.

### Transmission electron microscopy

Animals were deeply anesthetized and perfused intracardially with 0.01 M PBS, followed by 2% paraformaldehyde and 2% glutaraldehyde in 0.1 M cacodylate buffer. The cochleae were opened and flushed with the fixative for 2 h at room temperature. After washing, the specimens were post-fixed for 1.5 h in 2% osmium tetroxide in 0.1 M phosphate buffer and then were dehydrated through a graded ethanol series and embedded in Epon (Oken Shoji). Ultrathin sections of the cochleae were cut, stained with uranyl acetate and lead citrate, and examined by electron microscopy (HT-7700, Hitachi).

## Results

In this study, we performed a detailed analysis of cochlear GJPs using a mouse model of a major type of DFN3. These mice have severe sensorineural hearing loss together with decreased EP, fewer cytoplasmic extensions, and a reduction in the cytoplasmic volume and the number of mitochondria in the fibrocytes of the spiral ligament, although no abnormalities have been observed in other organs [4].

**Figure 1:**
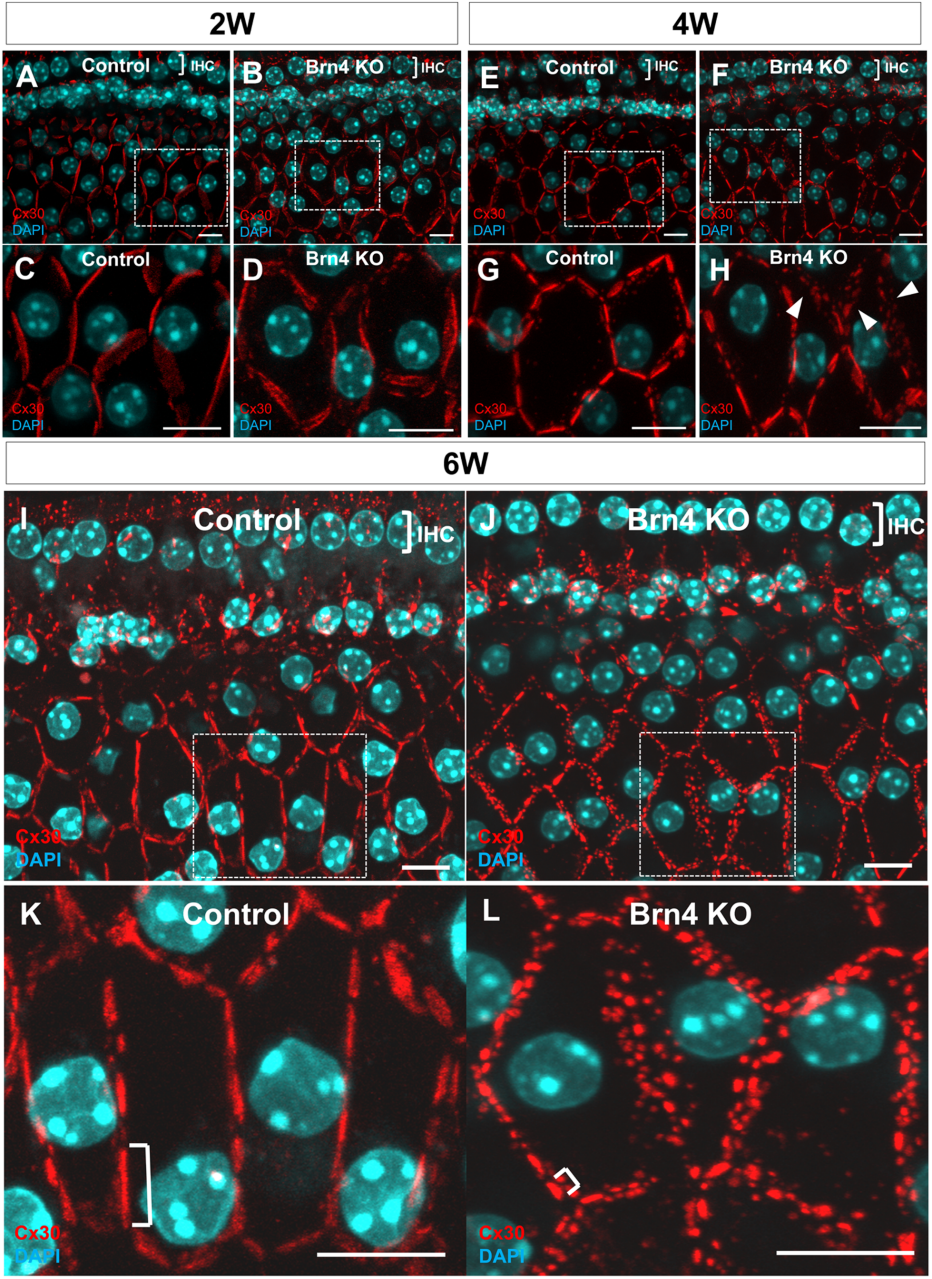
Brn4-deficient mice show a drastic disruption of GJPs with increasing age. Z-Stack images of cochlear inner sulcus cells from 2-, 4-, and 6-week-old littermate control and Brn4-deficient mice were obtained by confocal microscopy. GJPs were immunolabeled for Cx30 (red) (A–L). Nuclei were counterstained with DAPI (blue) (A–L). The positions of inner hair cells (IHCs) indicate the orientation of the images (A, B, E, F, I and J). High-magnification images of each boxed region in A, B, E, F, I and J are shown in C, D, G, H, K and L. The GJPs in 6-week-old Brn4-deficient mice were present as small spots around the cell-cell junction sites (J, L). In 2-week-old and 4-week-old littermate control and Brn4-deficient mice, the linear structure of the GJPs was present, although some plaques 4-week-old Brn4-deficient mice were disrupted and scattered (H, arrowheads). The lengths of the largest GJPs (brackets in K and L) along a single cell border were analyzed and are shown in Figure 4. Scale bars indicate 10 µm.

In littermate controls, gap junctions formed linear GJPs along the cell-cell junction sites with adjacent cells, and these formed pentagonal or hexagonal outlines of normal inner sulcus cells and border cells (Figure 1 A, C, E, G, I, K). The GJPs in 6-week-old Brn4-deficient mice did not show the normal linear structure but instead were present as small spots around the cell-cell junction sites (Figure 1 J, L). The gap junction units of the Brn4-deficient mice were substantially shorter than those in the control mice (Figure 1 I, K). In 2-week-old littermate control and Brn4-deficient mice, GJPs formed linear units around the cells (Figure 1 A–D). In 4-week-old littermate control and Brn4-deficient mice, the linear structure of the GJPs was present, although some plaques were disrupted and scattered in the cochleae of Brn4-deficient mice (Figure 1 E–H). In 6-week-old Brn4-deficient mice, a number of small GJPs that contained Cx30 were scattered around the cell-cell junction sites (Figure 1 J, L). The length of each GJP was notably shorter than those in the littermate controls (Figure 1 I, K).

**Figure 2:**
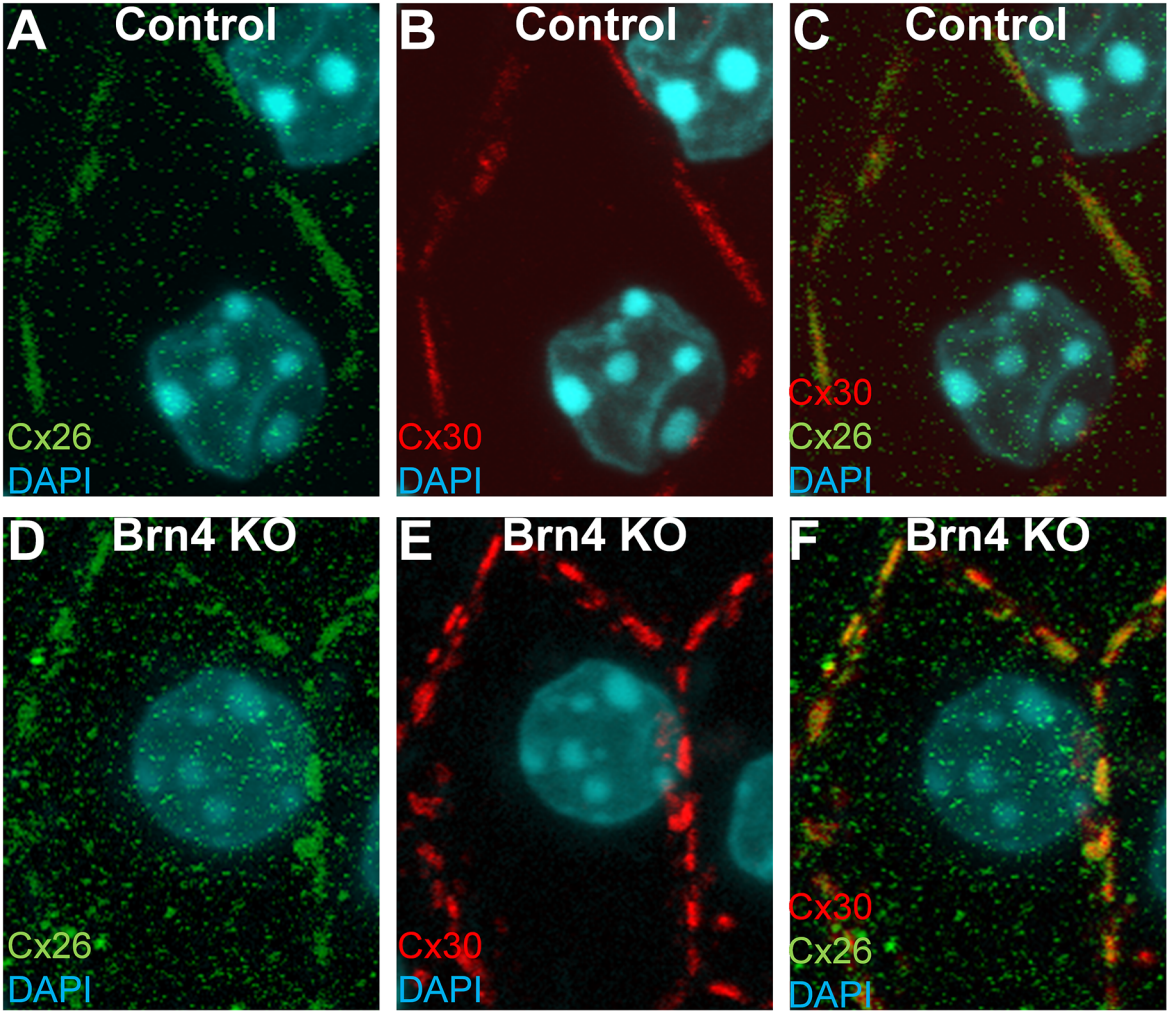
GJPs in Brn4-deficient cochleae are composed of Cx26 and Cx30 as in control littermates. Inner sulcus cells in cochlear whole-mount sections from a 6-week-old littermate control (A–C) and Brn4-deficient (knockout, KO) mouse (D–F). Images were obtained by confocal microscopy and z-stacking after immunolabeling for Cx26 (green) (A, D) and Cx30 (red) (B, E). Merged images are also shown (C, F). Nuclei were counterstained with DAPI (blue) (A–F). Bars indicate 10 µm.

In littermate controls, gap junctions were composed mainly of Cx26 and Cx30 (Figure 2 A–C). GJPs co-immunolabeled for Cx26 and Cx30 in inner sulcus cells were also observed in Brn4-deficient mice (Figure 2 D–F). Although there was a possibility that the Connexin-immunostaining might not indicate gap junction but hemichannel, the transmission electron microscopy showed the typical structures for gap junctions with inter-membrane layer also in Brn4-deficient mice as well as littermate controls (Figure S1).

**Figure 3:**
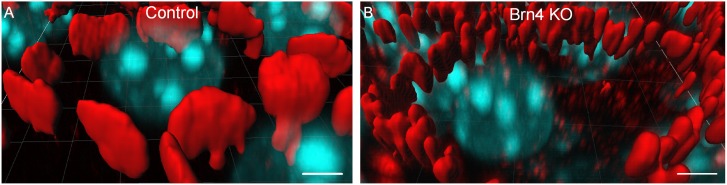
Three-dimensional reconstruction shows disrupted GJPs in Brn4-deficient mice. Three-dimensional images were reconstructed to show the detailed structure of GJPs from the *z*-stack images shown in Figure 1 I, J. GJP formation in adult (6w) cochleae from a littermate control (A) and a Brn4-deficient mouse (B). GJPs were immunolabeled for Cx30 (red), and nuclei were counterstained with DAPI (blue) (A, B). Scale bars indicate 5 µm.

**Figure 4:**
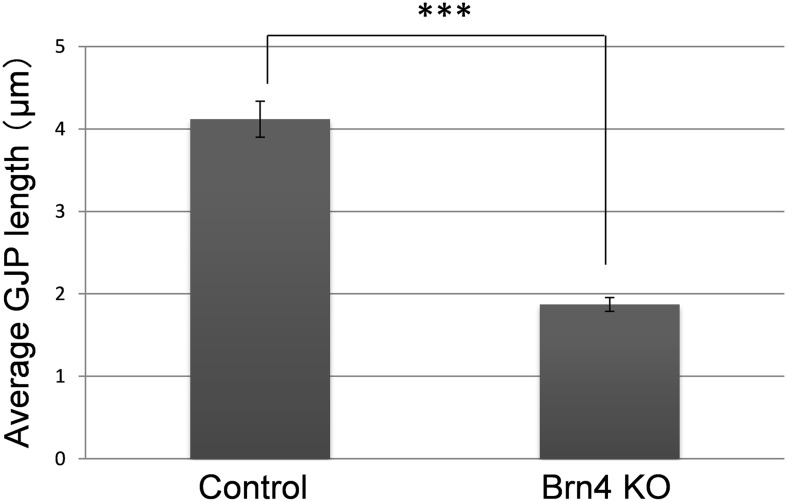
GJPs were significantly shorter in Brn4-deficient mice as compared with littermate controls at 6 weeks. The lengths of the largest GJPs (e.g., brackets in Figure 1 K and L) along a single cell border were measured in the *z*-stack confocal images and are expressed as the mean ± Standard Error (SE) (*n* = 81 GJPs per mouse type). ***P = 6.49×10^–16^ (Student’s *t*-test).

In the detailed analysis with three-dimensional graphic reconstruction of the GJP structure in the inner sulcus cells (ISCs; Figure 1 I, K), cochleae from adult littermate control mice showed large planar GJPs (Figure 3 A) at the cell borders that formed orderly pentagonal or hexagonal outlines around normal ISCs. In contrast, cochleae from Brn4-deficient mice (Figure 1 J, L) showed drastically fragmented small vesicle-like GJPs (Figure 3 B). To compare the size differences of the GJPs between Brn4-deficient mice and their littermate controls, the lengths of the largest GJPs with Cx30 (e.g., brackets in Figure 1 K, L) along a single cell border were analyzed in four Brn4-deficient mice and four control littermates. In 6-week-old Brn4-deficient mice, the average length of the GJPs was significantly shorter than that in the littermate controls (Figure 4).

**Figure 5:**
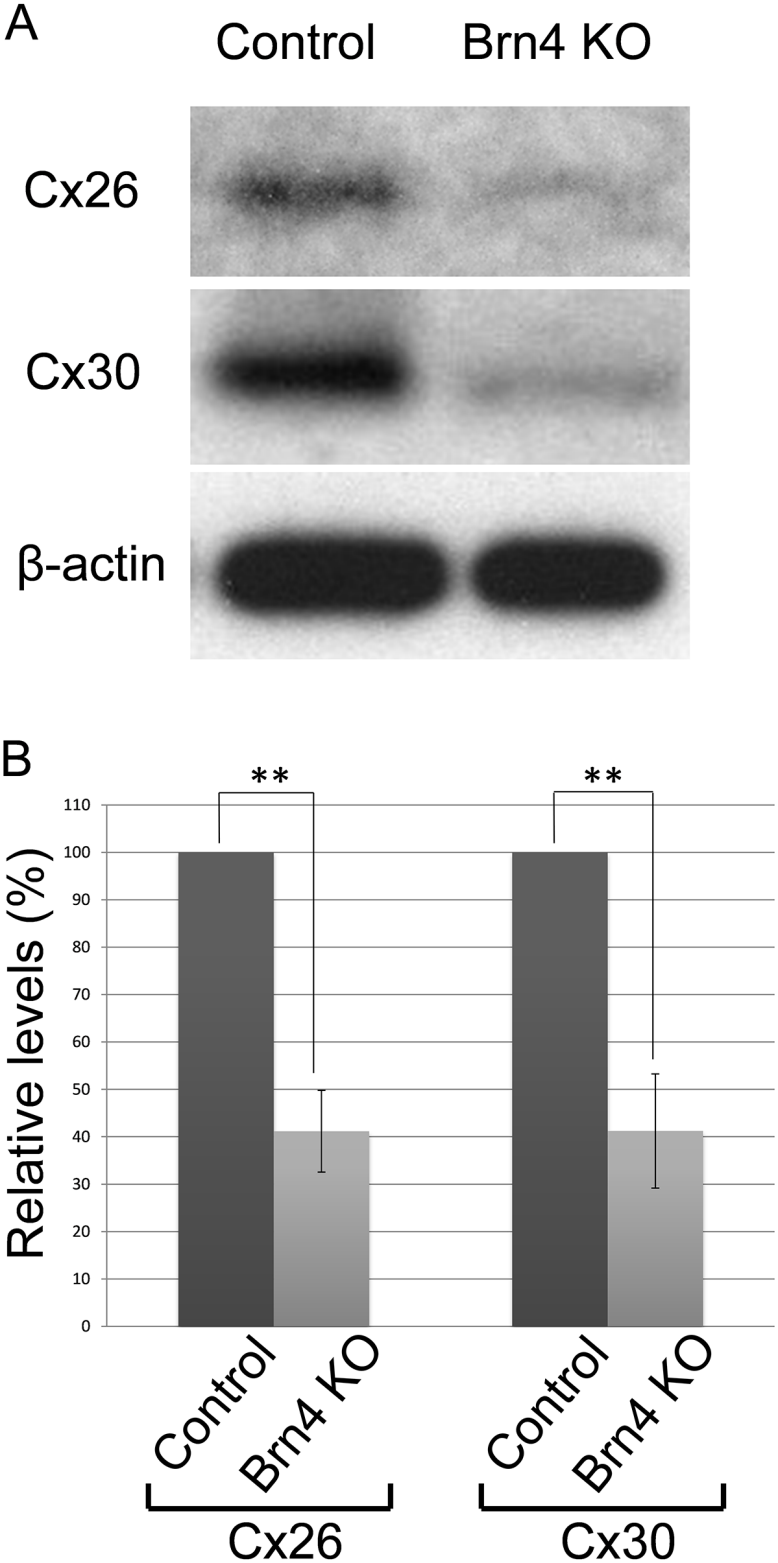
Gap junction protein levels were reduced in Brn4-deficient mice at 6 weeks. Western blot analysis of Cx26 and Cx30 was performed in 6-week-old Brn4-deficient mice and littermate controls. The level of Cx26 and Cx30 was normalized to the corresponding β-actin levels and expressed relative to the amount present in each of the littermate controls. Values represent the mean ± SE (*n* = 3 mice). ***P = 7.943×10^–3^ for Cx26 and ***P = 8.137×10^–3^ for Cx30, Student’s *t*-test.

**Figure 6:**
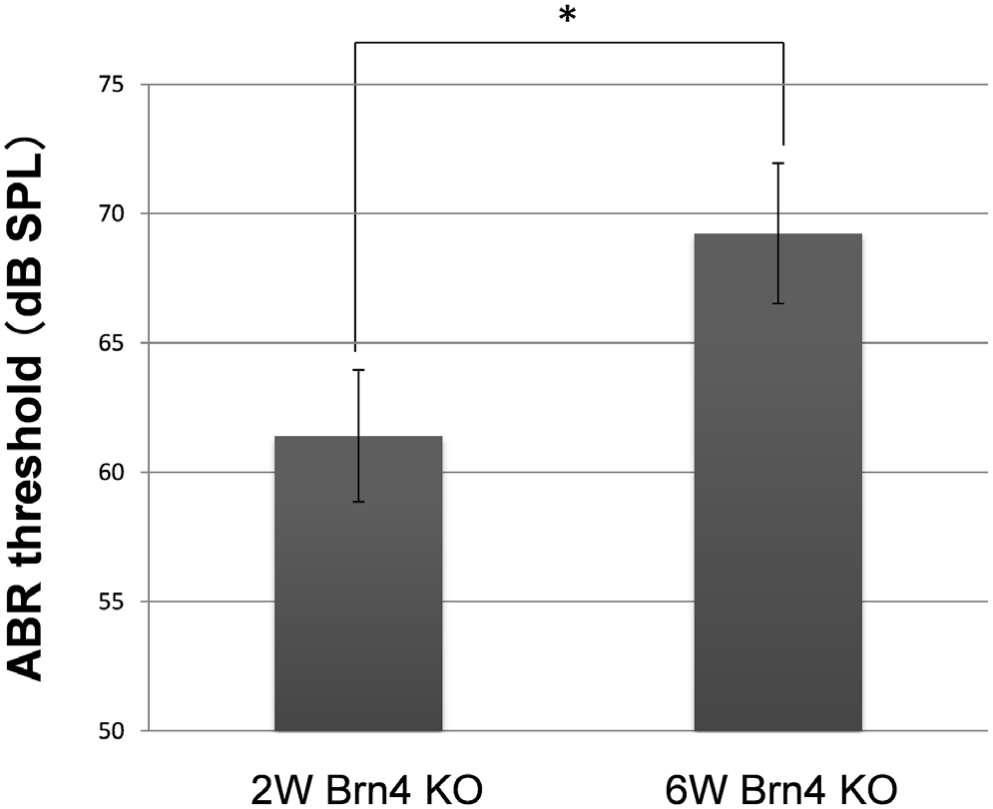
ABR thresholds were higher in Brn4-deficient mice at 6 weeks as compared with 2 weeks. ABR thresholds were analyzed in 2- and 6-week-old Brn4-deficient mice. In 2-week-old mice, the ABR threshold was 61.4±2.5 dB SPL (*n* = 5 mice), and in 6-week-old mice the ABR threshold was 69.2±2.7 dB SPL (*n* = 6 mice). Values represent the mean ± SE. *P = 3.40×10^–2^ (Student’s *t*-test).

In addition to GJP disruptions by the confocal analysis, 3D construction and their size analysis (Figure 1, 3 and 4), the significantly reduced levels of Cx26 and Cx30 in the Brn4-deficient mice (Figure 5) suggested that the gap junction macromolecular complex had degraded. Recently, we reported that assembly of the cochlear gap junction macromolecular complex requires connexin 26 [8]. In this paper, we demonstrated that cochlear gap junction plaque, macromolecular complex were mainly composed of Connexin 26 and Connexin 30, and this molecular complex were degraded together with the activation of endocytosis with upregulation of Caveolin proteins, in case of the Cx26-deficiency. It has also been reported in the other papers that the gap junction molecular complex were degraded via some proteasome pathways such as ubiquitination, autophagy, or endocytosis [9]–[11]. To assess the change in hearing function, ABR analyses were performed for 2- and 6-week-old male Brn4-deficient mice. ABR thresholds were significantly higher in the 6-week-old mice than in the 2-week-old Brn4-deficient mice (Figure 6, Figure S2). On the other hand, wild type mice in 2 and 6-week old showed normal ABR threshold (9.4±2.9 dB SPL, *n* = 5 for 2-week-old and 4.8±2.6 dB SPL, *n* = 7 for 6-week-old wild type mice).

## Discussion

In the present study, we examined the novel molecular pathology of hereditary deafness caused by Brn4 gene deficiency in a mouse model of DFN3. An abnormal morphology of the cell-to-cell adhesion of fibrocytes has been observed in 11-week-old Brn4-deficient mice [4]. At the spiral ligament, type IV and V otic fibrocytes are absent in these Brn4-deficient mice [4]. Cx26 expression is lost in type I and V fibrocyte regions of the spiral ligament [4]. In postnatal day 12 mice, Cx26 expression in type I and V fibrocyte regions is present in wild-type mice but not in Brn4-deficient mice [4]. In contrast to the spiral ligament, the cellular and molecular pathology of cochlear supporting cells has not been reported. In our previous study [4], [12], we have not identified any changes in connexin expression of the lateral wall fibrocytes in younger stage than 11-week old. However in the present study, we analyzed the subcellular expression of connexins with the 3D structure of GJP acquired from the z-scan confocal images of whole mount cochlea together with quantitative western blotting, and found the significant change in connexin expressions in postnatal development of Brn4-deficient mouse. Here we showed that Brn4 substantially contributes to cochlear gap junction properties to maintain the proper EP in cochleae, similar to connexin-related deafness. This finding suggests that the hearing loss was caused by an effect on connexins such as Cx26 and Cx30, which represent the proteins that are most frequently mutated in hereditary deafness. The disruption of GJPs observed after 4 weeks in the Brn4-deficient mice was also observed in our two Cx26 mutant mice [8]. This suggests that Brn4 expression is directly or indirectly involved in gap junction formation in ISCs. The reduction in the GJP area may then abolish the proper biophysical potential needed for intercellular communication in the cochlea, resulting in a significant elevation of the ABR threshold (Figure 6). This may explain why Brn4-deficient mice showed significantly decreased EPs in our previous study [4]. In that study, EP was not absent but reduced from 85 mV (wild-type mice) to 38 mV in 11-week-old male Brn4 deficient mice. Although we have not measured EP before 11 weeks old and the biochemical or structural changes of GJP have not been detected around 2-week-old mice, there is a possibility that the EP was not absent but already lower than the control level (∼+80 mV) in 2-week-old, and then progressively reduced to around 38 mV or maintained low EP level by 11 weeks old, because the ABR data in the present study (Figure 6) indicated the hearing loss already in 2-week-old Brn4-KO mice. Currently, the downstream targets of POU3F4, including gap junction genes and their proposed transcription factors in the cochlear supporting cells, are unknown. Only a few genes have been reported to be involved in fibrocyte differentiation including Otospiralin (Otos) [13] and Tbx18 [14], although Song et al. reported that neither Otos nor Tbx18 expression was affected in the spiral ligament of Brn4-deficient mice [15]. Although the entire molecular pathway between Brn4 and the cochlear gap junction has not yet been worked out, our data clearly demonstrated that POU3F4 affects the expression and assembly of the gap junction proteins Cx30 (GJB6) and Cx26 (GJB2) in cochlear supporting cells. GJP degradation along with a decrease in the component connexins may explain the decrease in the EP and the severe hearing loss in Brn4-deficient mice and DFN3 patients. Furthermore, gene therapy targeting cochlear gap junction proteins, such as Cx26 gene transfer, may also be effective for DFN3 patients.

## Supporting Information

Figure S1 **Ultrastructures of gap junctions in Brn4 deficient mice by Transmission Electron Microscopy (TEM).** Ultrathin sections of ISCs showed the gap junctions in 6-week-old Brn4 deficient mice (B) with an inter-membrane layer between both clearly visible plasma membranes that maintains the same distance (2–4 nm) in both mutant mice and control littermates (A). There were no obvious differences between Brn4 deficient mice (B) and control mice (A). Inset shows high-magnification image of boxed area, which contains a gap junction. Bars indicate 100 nm.
(DOCX)

Figure S2 **ABR waveforms of control (+/Y) and Brn4 deficient (−/Y) males at 6 weeks old of age, measured at 60-, 70- and 80-dB SPL.**
(DOCX)
