# Supplementary material for: Deficiency of Transcription Factor Brn4 Disrupts Cochlear Gap Junction Plaques in a Model of DFN3 Non-Syndromic Deafness
Source: PLoS One. 2014 Sep 26;9(9):e108216. doi: 10.1371/journal.pone.0108216 (PMC4178122; doi:10.1371/journal.pone.0108216)
Supplement: Figure S1 — Ultrastructures of gap junctions in Brn4 deficient mice by Transmission Electron Microscopy (TEM). Ultrathin sections of ISCs showed the gap junctions in 6-week-old Brn4 deficient mice (B) with an inter-membrane layer between both clearly visible plasma membranes that maintains the same distance (2–4 nm) in both mutant mice and control littermates (A). There were no obvious differences between Brn4 deficient mice (B) and control mice (A). Inset shows high-magnification image of boxed area, which contains a gap junction. Bars indicate 100 nm. (DOCX) [file pone.0108216.s001.docx]

**Figure S1**

**
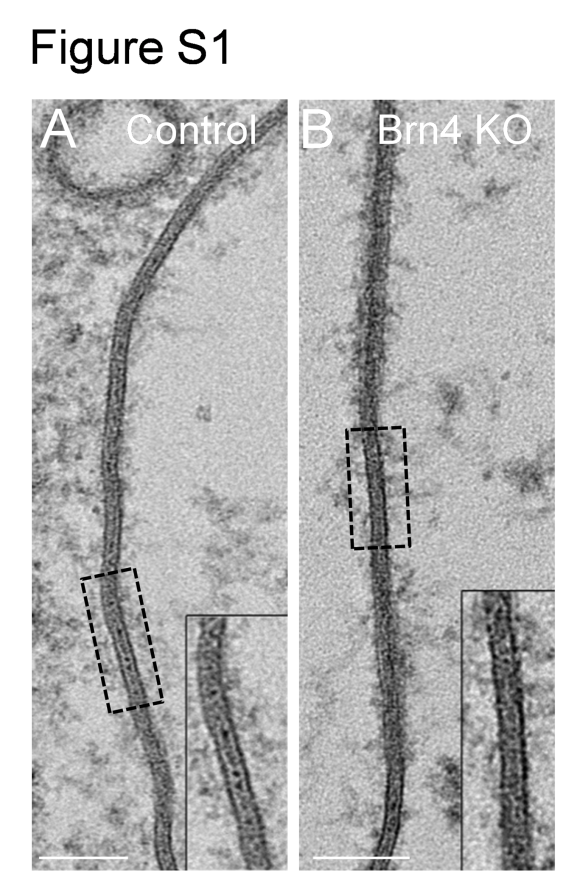
**

**Figure S1 Ultrastructures of gap junctions in Brn4 deficient mice by Transmission Electron Microscopy (TEM).** Ultrathin sections of ISCs showed the gap junctions in 6-week-old Brn4 deficient mice (B) with an inter-membrane layer between both clearly visible plasma membranes that maintains the same distance (2–4 nm) in both mutant mice and control littermates (A). There were no obvious differences between Brn4 deficient mice (B) and control mice (A). Inset shows high-magnification image of boxed area, which contains a gap junction. Bars indicate 100nm.
