# Supplementary material for: Deficiency of Transcription Factor Brn4 Disrupts Cochlear Gap Junction Plaques in a Model of DFN3 Non-Syndromic Deafness
Source: PLoS One. 2014 Sep 26;9(9):e108216. doi: 10.1371/journal.pone.0108216 (PMC4178122; doi:10.1371/journal.pone.0108216)
Supplement: Figure S2 — ABR waveforms of control (+/Y) and Brn4 deficient (−/Y) males at 6 weeks old of age, measured at 60-, 70- and 80-dB SPL. (DOCX) [file pone.0108216.s002.docx]

**Figure S2**


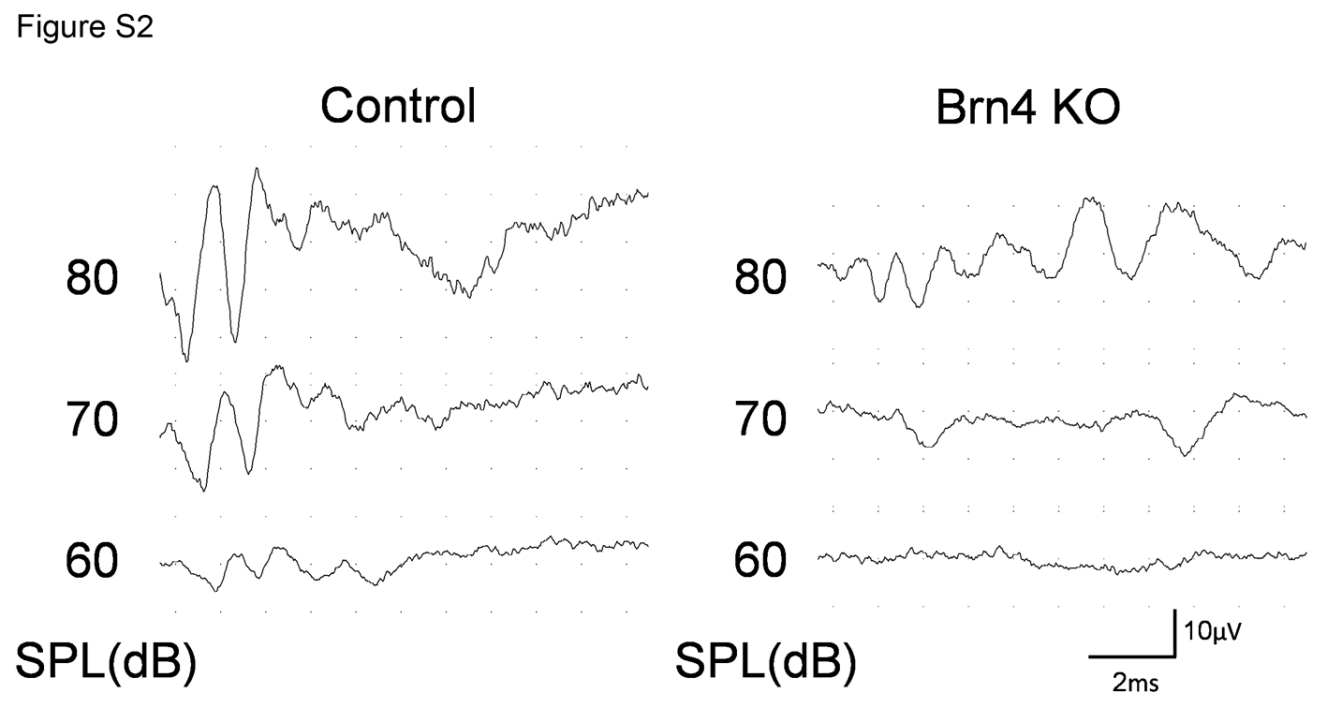


**Figure S2. ABR waveforms of control (+/Y) and Brn4 deficient (-/Y) males at 6 weeks old of age, measured at 60-, 70- and 80-dB SPL.**
